# Exploring the metabolic changes of *Ceratitis capitata* Vienna 8 strain across three developmental stages through probiotic larval diet supplementation

**DOI:** 10.1371/journal.pone.0313894

**Published:** 2024-12-02

**Authors:** M. Msaad Guerfali, K. Charaabi, H. Hamden, O. Zidi, M. Hamdi, S. Fadhl, S. Kouidhi, A. Cherif, A. Mosbah

**Affiliations:** 1 Laboratory of Biotechnology and Nuclear Technologies LR16CNSTN01, National Center of Nuclear Sciences and Technologies, Ariana, Tunisia; 2 Laboratory of Biology and Bio-Georessources LR11ES31, Higher Institute of Biotechnology of Sidi Thabet, University of Manouba, Ariana, Tunisia; University of Carthage, TUNISIA

## Abstract

The Sterile Insect Technique (SIT) is revolutionizing pest control through its environmentally friendly approach, which involves rearing and sterilizing male insect pests using ionizing radiation and subsequently releasing them into the field to mate with wild females without producing offspring. Recent research has unveiled a groundbreaking enhancement in the quality of released *Ceratitis capitata* (medfly) males through the addition of probiotics to their larval diet. To thoroughly examine the impact of probiotic supplementation on the medfly larval diet, we conducted an in-depth analysis with GC-MS from medfly extract supplemented with probiotic *Enterobacter* sp. strain. The supplementation revealed a notable difference in the metabolomic signature compared to non-supplemented extract across all medfly life stages. We identified 37 known metabolites for all the stages, 12 of them were identified as biomarkers for the larval stage, 12 for the pupal stage, and 13 for the adult stage exhibiting crucial activities such as anti-bacterial, anti-fungal, and sexual and aggregation pheromone. These findings underscore the considerable potential of SIT combined with probiotic supplementation for enhancing sustainable pest control strategies worldwide.

## Introduction

The World Health Organization (WHO), defines probiotics as living organisms which, when administered in adequate amounts, confer health benefits to the host [1]. Although their primary applications traditionally focused on humans, livestock, fish and poultry, the past decade has seen a growing interest in the use of probiotics to mass-reared insects with promising potential. Probiotics hold significant promise for insect rearing facilities and biocontrol. In particular, the Sterile Insect Technique (SIT), which involves the mass rearing of target pest species, sterilization through irradiation, and release into infested areas to suppress wild populations, may benefit from the incorporation of probiotics [2]. While not a recent innovation, the SIT continues to evolve for various pest species and is being adopted by multiple countries, with some facilities achieving substantial production of sterile insects [3]. Efforts to refine this approach are exemplified through the incorporation of probiotics in the mass production of insects as evidenced by supplementation trials conducted on the diet of the Mediterranean fruit fly, *Ceratitis capitata* (Weideman) (medfly). Medfly is among the pests of economic importance subject to extensive mass rearing, with bioproduction facilities employing specific laboratory lines that may exhibit lower competitiveness compared to wild strains [3]. The medfly Vienna 8 strain, enables the selective release of irradiated sterile males into the wild by facilitating the early separation of females during the rearing process. Supplementation of the diets of both adult and larval medflies with probiotics such as *Klebtiella* sp., *Enterobacter* sp., *Citrobacter* sp., has led to enhancements in certain fitness traits of the produced insects. Specifically, the incorporation of *Klebtiella oxytoca* into the adult diet has been associated with improved mating competitiveness and reduced receptivity [4,5]. Pioneering work conducted by Hamden et al. [6] further explores this dynamic as they were the first to apply a cocktail of probiotics to the larval diet, resulting in positive outcomes such as improved weight and other parameters. However, some critical comments have been raised regarding the origin of the added bacteria [7]. Augustinos et al. [7] enriched the larval medium with a probiotic bacterium *Enterobacter* sp., isolated from the medfly gut, which resulted in improved pupal and adult productivity, along with a shortened rearing duration. Subsequent studies by Kyristis et al. [8,9] focused on incorporating both live and autoclaved probiotics into the larval diet, with findings suggesting that inactivated bacteria might serve as a complete substitute to the brewer’s yeast as a protein source in the medfly larval diet. More recently, Hamden et al. [10] implemented a probiotic selection strategy for the ‘*in-vitro*’ and ‘*in-vivo’* assessment of probiotic properties. The mechanisms of action of probiotics and their effects on the medfly were comprehensively reviewed by Msaad Guerfali et al. [11].

These lines of evidence suggest a favorable role of probiotics following dietary supplementation in medflies. However, further investigations are needed to elucidate their effects on host metabolism, changes in gut microbiota structure, and their capacity to colonize the gut and interact with local microbiome niches.

In the present study, our objective was to leverage the substantial potential of entometabolomics to examine the influence of probiotic bacterial supplementation on the metabolomic profiles of male medflies at various developmental stages. We utilize advanced high-throughput methods to accurately identify metabolites, aiming to discern the most advantageous compounds during different life stages of the medfly.

## Materials and methods

### Stock fly and maintenance

The Mediterranean fruit flies were from a colony of the VIENNA 8 genetic sexing strain (GSS) maintained in the Tunisian Medfly rearing facility at the National Center for Nuclear Science and Technology (CNSTN). This strain has two mutations with two markers *wp* and *tsl* on Y-autosome 5 (D53 inversion) producing wild-type (brown pupae) males and mutant (white pupae) females; with an increased genetic stability. Adult flies were fed with sugar:yeast (3:1) and water. The larval diet contained wheat bran as a bulking agent (28%), torula yeast as a protein source (7%), sugar as a phagostimulant and carbohydrate source (14%), and water (50%). All experiments were carried out under laboratory conditions (23°C ±1°C and 60% RH) [12,13].

### Probiotic supplementation

*Enterobacter* sp. was isolated from the guts of adult Tunisian wild strain medflies. This strain was selected as a potential probiotic candidate for inclusion in larval diets as documented by Hamden et al. [10]. Bacterial cells were concentrated to an optical density equal to 0.3 at 550 nm in Luria-Bertani (LB) medium agar. These cells were then incorporated into 100 g of larval diet for each treatment [6]. Each treatment was replicated four times. The control diet received a supplementation of an equivalent volume of LB broth devoid of bacterial cells.

### Irradiation procedure

The collected pupae originating from different diets underwent irradiation during the pre-emergence virgin phase, precisely two days before emergence (8- day-old) utilizing the Cobalt-60 irradiator. A locally designed irradiation apparatus for Medfly comprises 4 rotating disks that allow the rotation of canisters within the radiation field [14]. The axis of rotation is vertical and runs parallel to the irradiator source pens. Male pupae resulting from probiotic supplementation were subjected to a radiation dose of 90 Gy [15,16]. Concurrently, control pupae without probiotic supplementation were also irradiated at the identical dose.

### Metabolomics study

#### Sample preparation

Five grams of each medfly stage (larvae, pupae, and adult), were crushed in a mortar and homogenized in 25 ml of ethylacetate under ice-cold conditions. A first liquid-solid extraction was carried out at 4000 rpm for 15 mn for the solid fraction, and 25 ml of acetone was added. A second centrifugation yielded a second supernatant to which 25 ml of methanol was added to the pellet. After a centrifuge, the third supernatant was retained. The three supernatants were evaporated at 60°C using a rotavapor to obtain three solid fractions (200 mg, 250 and 500 mg, respectively) [17].

Regarding the probiotic strain, a single colony of the bacteria *Enterobacter* sp. was enriched in LB for 24 h at 37°C. Serial dilutions were then carried out from this stock solution (10^−1^ to 10^−20^), and each dilution was plated on LB agar (100 μl). Three replicates were prepared for each dilution and colonies were counted 24 h after incubation at 37°C for each dilution. The optical density was measured in a spectrophotometer at 600 nm. One milliliter of each dilution was withdrawn, sonicated (3 mn at 30% amplitude), and subjected to centrifugation. The supernatant was kept for the extraction of secondary metabolites. Two milliliters of the ethylacetate were added to the supernantent for all the dilutions, and homogenized. A first centrifugation was performed to obtain the first supernatant. Two milliliters of acetone were added to the pellet for all dilutions and, after homogenization and centrifugation, the second supernatant was retained. Finally, 2 ml of methanol were added to the pellet, and, after homogenization and centrifugation, the third supernatant was retained [17]. The three supernatants were evaporated in a Rota vapor at 60°C (200, 250, and 500 mg, respectively).

#### GC-MS sample preparation, derivatization, and spectral acquisition

Metabolic profiling was performed on Agilent GC 7890BMS 240 ion trap gas chromatography (GC) system, equipped with an MS detector and HP-5MS capillary column (30 m × 250 μm, film thickness 0.25 μm). The injector temperature was set at 280 °C and GC oven temperature was initiated at 40 °C for 2 min, followed by an increase of 5 °C/min to 250 °C, where it was maintained for 20 min. The analysis was carried out in full scan mode for 60 min, using helium as the gas carrier at a flow rate of 1 ml/min. Subsequently, 1 μl of supernatant from each sample was injected in split mode with an ionization range from 50 to 1000 mV. Metabolite identification was achieved by comparing their mass spectra with those referenced in the Library (NIST).

#### Identification and comparison of volatile compounds

Mass spectral data processing and metabolite identification were performed using the Automated Mass Spectral Deconvolution and Identification System (AMDIS) (AMDIS-version 2.71, 2012) and the National Institute of Standards and Technology (NIST) (version 2.0, 2011) database. The detected metabolite peaks underwent validation using three components within NIST; these were a Match of > 800, a 90% probability of a match to NIST library standards, and a head-to-tail comparison of the fragments. Further metabolite validation was performed by matching experimental tandem MS spectra, retention time, and Cas number of the metabolic features against PubChem library (https://pubchem.ncbi.nlm.nih.gov/). A compound was deemed present when meeting these three criteria. This process provides a relative ion abundance; no units of ion abundance were available. Compounds with a similarity index exceeding 80% were considered as a potential biomarker, hence, compounds that were found in less than 20% of the entire sample cohort were removed from further analysis [18].

#### Data analysis

We attempted to analyze the metabolic profile among the different groups by performing multivariate statistical analysis using SIMCA 16. Initially, the principal component analysis (PCA) was carried out to identify any outliers within the data set. Then, an orthogonal partial least squares-discriminant analysis (OPLS-DA) was applied to optimize the separation between the different groups. The model robustness was evaluated with the R^2^Y (fraction of variance), the Q^2^ (model predictability), and p-values. Close to 1, R^2^Y and Q^2^ values indicate an excellent model, whereas low values are indicative of model over-fitting. The variable importance in the projection (VIP) values of all peaks from OPLS-DA were taken as coefficients for peak selection to find the features significantly differentiating between groups. The statistical model was tested for robustness by a Y-permutation performed on PLS-DA, which confirmed the observed metabolic variations. The statistical model was tested for robustness by a Y-permutation performed on PLS-DA, which confirmed the observed metabolic variations and by the use of a CV-ANOVA from SIMCA-P 16 (analysis of variance in the cross-validated residuals of a Y variable). A hierarchical cluster analysis heatmap was obtained using the ward clustering algorithm and Euclidean distance calculation to further confirm the results of PLS-DA and to show the distribution of metabolites among the groups. The heatmap analyses were performed using the metaboAnalystv5.0.

#### Selection of biomarkers

We constructed Receiver Operating Characteristic (ROC) curves to check the accuracy of the model, using MetaboAnalyst v5.0. A forward stepwise logistic regression model was constructed to design the best metabolite combination. ROC curves were used to test the accuracy of the model. The global performance of each biomarker was evaluated using the Area Under the Curve (AUC) and the determination of sensitivity and specificity [19]. The data obtained was subjected to an unpaired non-parametric test (Wilcoxon rank-sum test, also known as Mann-Whitney U-test) within MetaboAnalyst and false discovery rates (FDR), calculated by the SAM (Significant Analysis of Microarray) analysis which is essentially used for microarray data, but it is also used for metabolomic data (GC-MS; LC-MS and NMR compounds), were calculated to discover if those metabolites are significantly different between groups [20]. All features with FDR values below 0.05 indicated that these features can indeed be regarded as potential “biomarkers”.

## Results

### Analysis of medfly metabolomic and biochemical profile by GC-MS

Samples underwent analyses using a GC–MS approach, which has been shown to make a comprehensive metabolic fingerprint with good analytical characteristics in insect studies. This method demonstrated its effectiveness as a suitable tool for investigating the metabolic and its biochemical modifications following probiotic administration. In the aforementioned GC–MS analysis, the metabolites were identified using the NIST library based on three criteria: match score of greater than 80%, a probability of a match to NIST library standards of at least 90%, and a head-to-tail comparison of the mass spectra fragments. After excluding the non-endogenous metabolites, such as solvents, and reagents, as well as those with missing values, a total of 150 metabolite features, following all the extraction solvents, were detected across the different life stages both with and without probiotic supplementation. This set included both hydrophilic and hydrophobic metabolites, and all 150 metabolite features were used for the following multivariate statistical analysis.

### Assessment of metabolomic data

**Fig 1:**
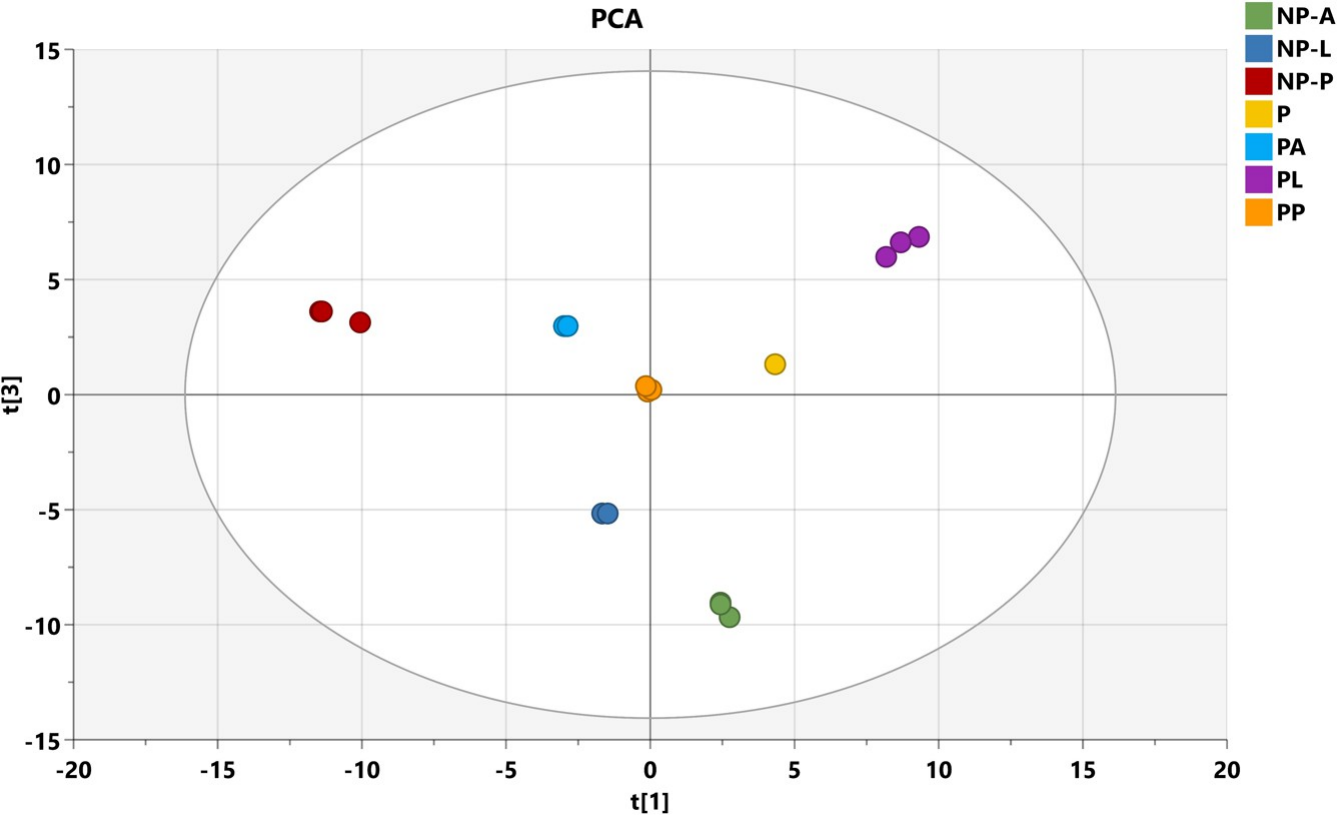
The score plot of PCA based on the GS-MS data of the medfly stages samples supplemented or not with probiotics. *P* is the probiotic strain *Enterobacter* sp., *A* the control adult, *L* the control larvae, *P* is the control pupae, *PA* is the probiotic adult, *PL* is the probiotic larvae, and *PP* is the probiotic strain. The Hoteling ellipse indicates a 95% confidence interval. *NP*: Non-probiotic.

The application of PCA and OPLS-DA models to the medfly stages, including treated, non-treated, and probiotic strains, revealed robust and significant separations among the different treatments. In the principal component analysis (PCA), we were able to differentiate the metabolic profiles of the probiotic strain, probiotic stages, non-probiotic stages, and even the probiotic strain itself using the first two principal component (PC) scores. This highlights the significant impact of probiotic supplementation on the metabolomic profiles of the medfly males at various developmental stages (Fig 1). The abscissa of the PCA score chart represents the first principal component, PC1, denoted as t [1], while the ordinate represents the second principal component, PC2, denoted as t [3]. The clustering of biological replicates from the same time point suggests good repeatability for each treatment. The R2X value of the PCA model, representing the explained variance for the groups, was 0.738. All groups were within the Hoteling ellipse of 95% confidence, indicating that the analyzed samples contained no outliers. The established model had good fitting accuracy and could be reliably used to explain the metabolic differences between the studied groups.

**Fig 2:**
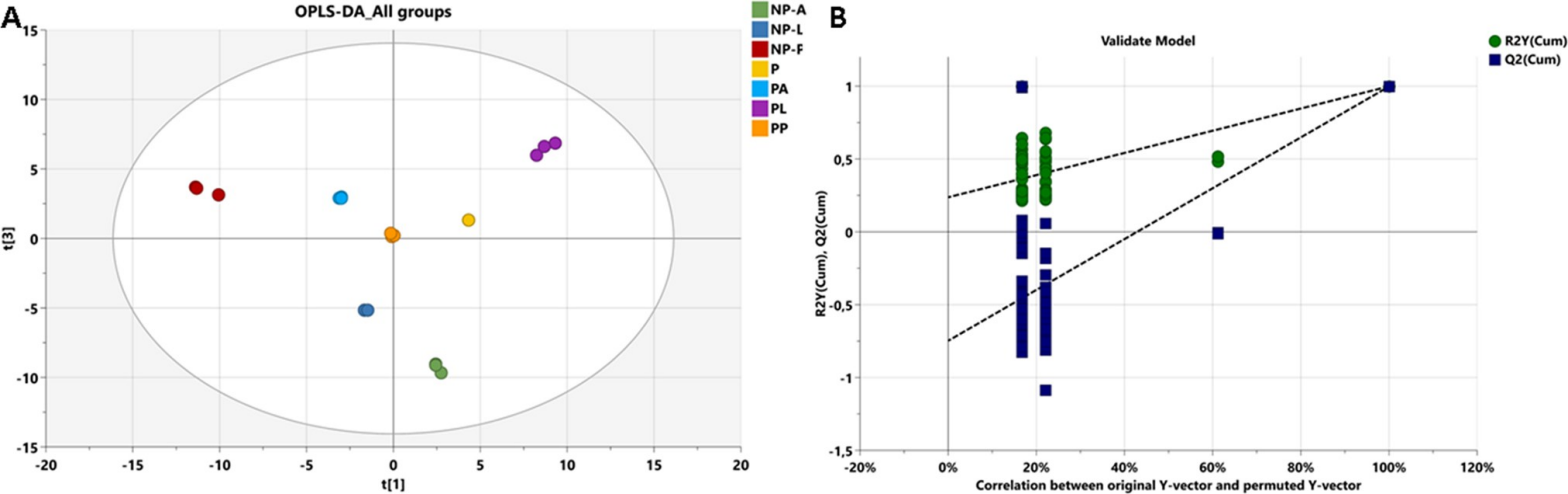
OPLS-DA model analysis of metabolomic data and permutation test. (A): Data for OPLS-DA model analyses for all groups with R^2^X = 0.97; R^2^Y = 0.998; Q^2^ = 0.997 and p-value = 0.003 (B): Permutation test validation of OPLS-DA model for all groups with 100 permutation tests. Green circles and blue squares represent R2 and Q2, respectively. Intercepts R2 = (0.0, 0.202), Q2 = (0.0, -0.759).

To further elucidate the significant metabolic differences between the various medfly life stages, with and without probiotics, we performed comparative orthogonal partial least squares discriminant analyses (OPLS-DA). The model comprised three orthogonal components and one predictive component calculated yielding a score plot that distinctly separated the different groups. The two-dimensional (2D) OPLS-DA score plots of the biochemical profiles among the different medfly life stages with and without probiotics (Fig 2A), revealed clear differentiation between all groups. The model exhibited robust fitness and predictability (R2X = 0.97; R2Y = 0.99 and Q2 = 0.997).

To validate the model, a permutation test with n = 100 was performed (Fig 2B). Furthermore, the CV-ANOVA test was employed to assess the statistical significance of differences within the OPLS-DA model between the two groups, yielding a p-value of 0.003, indicating highly significant differences between the groups within the model.

### Impact of the probiotic supplementation on the metabolic profile of medfly

**Fig 3:**
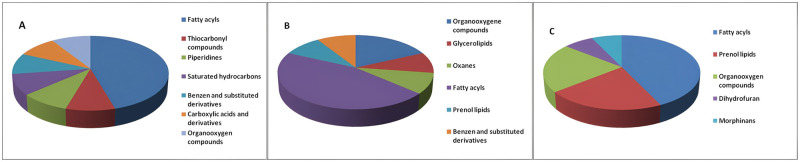
Metabolite classification. Identified metabolites for each stage (A) Larvae, (B) Pupae, and (C) Adults are categorized according to chemical class and the number of metabolites per class is significantly regulated after probiotic supplementation of the different medfly life stages.

Following different solvent extractions, the identified metabolites showed diverse chemical characteristics with varying concentrations between the three medfly life stages (Fig 3). Fatty acyls or lipids were found to be the major metabolite group in larvae, pupae, and adults, accounting for 45.45%, 41.67%, and 42.85% of the DEMs (Differentially Expressed Metabolites), respectively (Fig 3A–3C). Additionally, organooxygen compounds dominated the metabolite group in pupae, accounting for 41.67%. Notably, other metabolites such as glycerol lipids and prenol lipids were also detected, highlighting the complexity of metabolic pathways and the biochemical diversity present in the medfly at different life stages.

**Fig 4:**
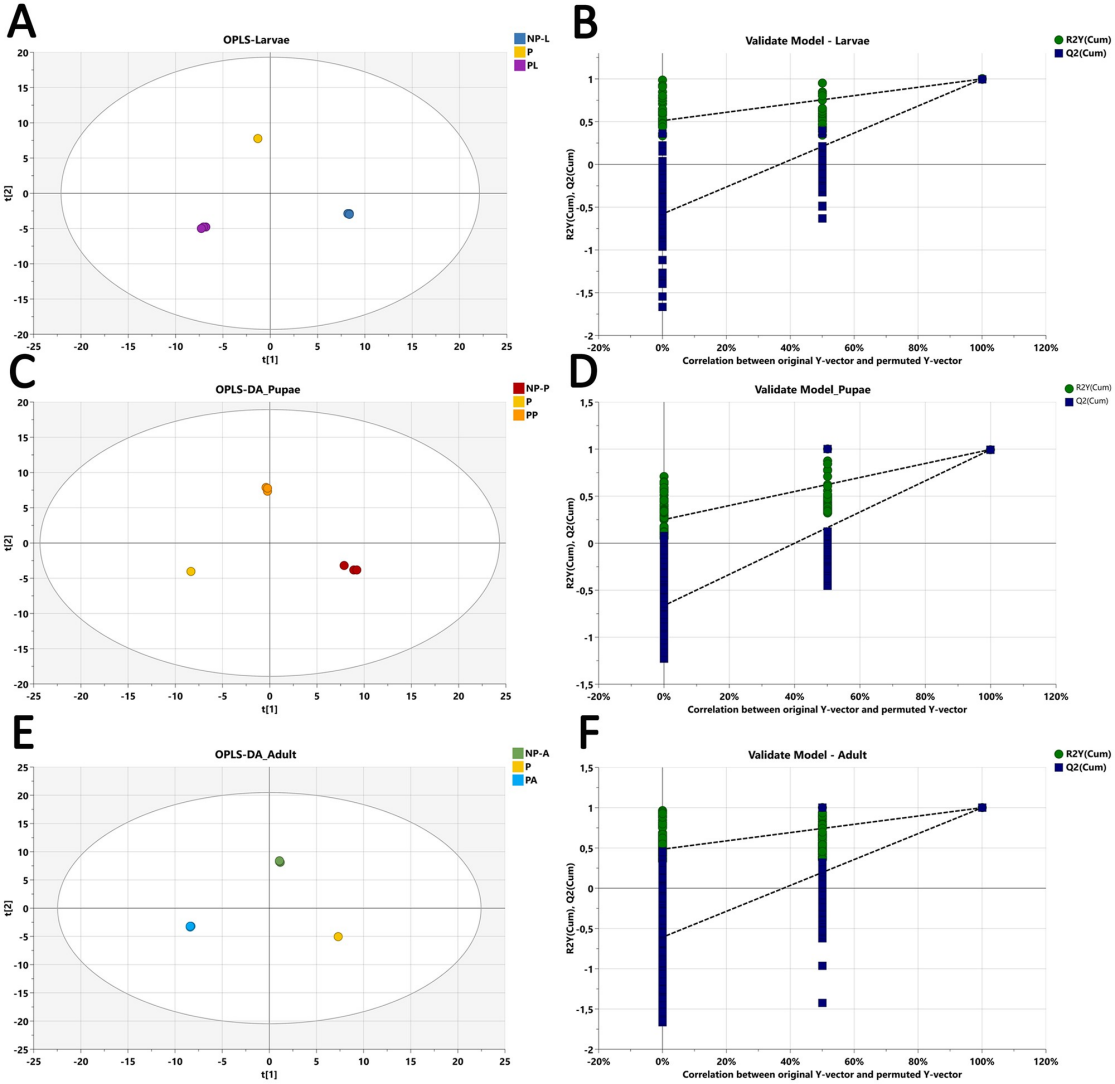
OPLS-DA model analysis of metabolomic data and permutation test. (A): Data for OPLS-DA model analyses comparing larvae with probiotic to larvae without probiotic with R^2^X = 0.974; R^2^Y = 1 and Q^2^ = 0.989 and p-value = 3.14e-07 (B): Permutation test validation of OPLS-DA model for larvae with 100 permutation tests. Green circles and blue squares represent R2 and Q2, respectively. Intercepts R2 = (0.0, 0.512), Q2 = (0.0, -0.579). (C): Data for OPLS-DA model analyses comparing pupae with probiotic to pupae without probiotic with R^2^X = 0.975; R^2^Y = 0.998; Q^2^ = 0.997 and p-value = 6.72e-09 (D): Permutation test validation of OPLS-DA model for pupae with 300 permutation tests. Green circles and blue squares represent R2 and Q2, respectively. Intercepts R2 = (0.0, 0.251), Q2 = (0.0, -0.664). (E): Data for OPLS-DA model analyses comparing medfly adults with probiotics to adults without probiotics with R^2^X = 0.981; R^2^Y = 1; Q^2^ = 1 and p-value = 3.28e-09 (F): Permutation test validation of OPLS-DA model for larvae with 999 permutation tests. Green circles and blue squares represent R2 and Q2, respectively. Intercepts R2 = (0.0, 0.485), Q2 = (0.0, -0.608).

To further investigate the probiotic supplementation on the medfly metabolomic profile, a pairwise analysis was conducted to compare each life stage with and without probiotics. The metabolomic and biochemical signatures revealed significant changes in response to probiotic administration through the different medfly life stages. The 2D OPLS-DA plots demonstrated clear separation between medfly larvae with and without probiotics (R^2^X = 0.974; R^2^Y = 1 and Q^2^ = 0.989) (Fig 4A), between pupae without and pupae with probiotics (R^2^X = 0.975; R^2^Y = 0.998; Q^2^ = 0.997) (Fig 4C), and between adults without probiotics and adults after probiotic supplementation (R^2^X = 0.981; R^2^Y = 1; Q^2^ = 1) (Fig 4E). These results suggest that probiotic supplementation alters the biochemical profile of medflies among all the life stages.

The robustness of our statistical models was tested using permutation tests (Fig 4B, 4D and 4F). Furthermore, the CV-ANOVA test was performed to assess the statistical significance of the differences among the various groups in the OPLS-DA models, yielding scores of p-values of 3.14e^-07^ for larvae, 6.72e^-09^ for pupae, and 3.28e^-09^ for adults. These results indicate highly significant differences between the groups within the models.

**Fig 5:**
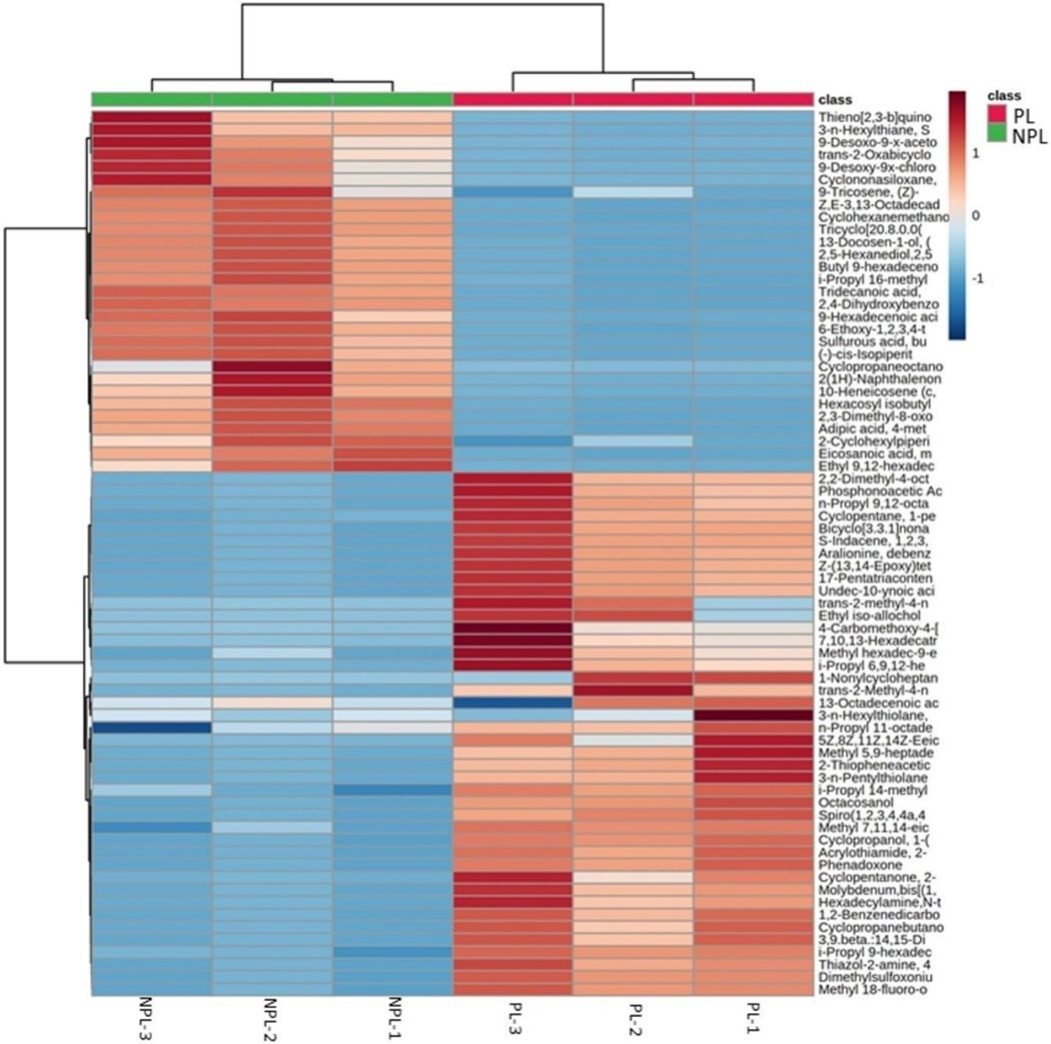
Hierarchical cluster analysis of medfly larvae metabolic biomarkers under probiotic supplementation (X-axis). The Y-axis represents individual metabolites that were identified and showed changes in abundance with respect to preceding and following time points (p < 0.05). Normalized signal intensities are visualized as a color spectrum in the heat maps. Red and blue represent high and low expression, respectively, of metabolites.

**Fig 6:**
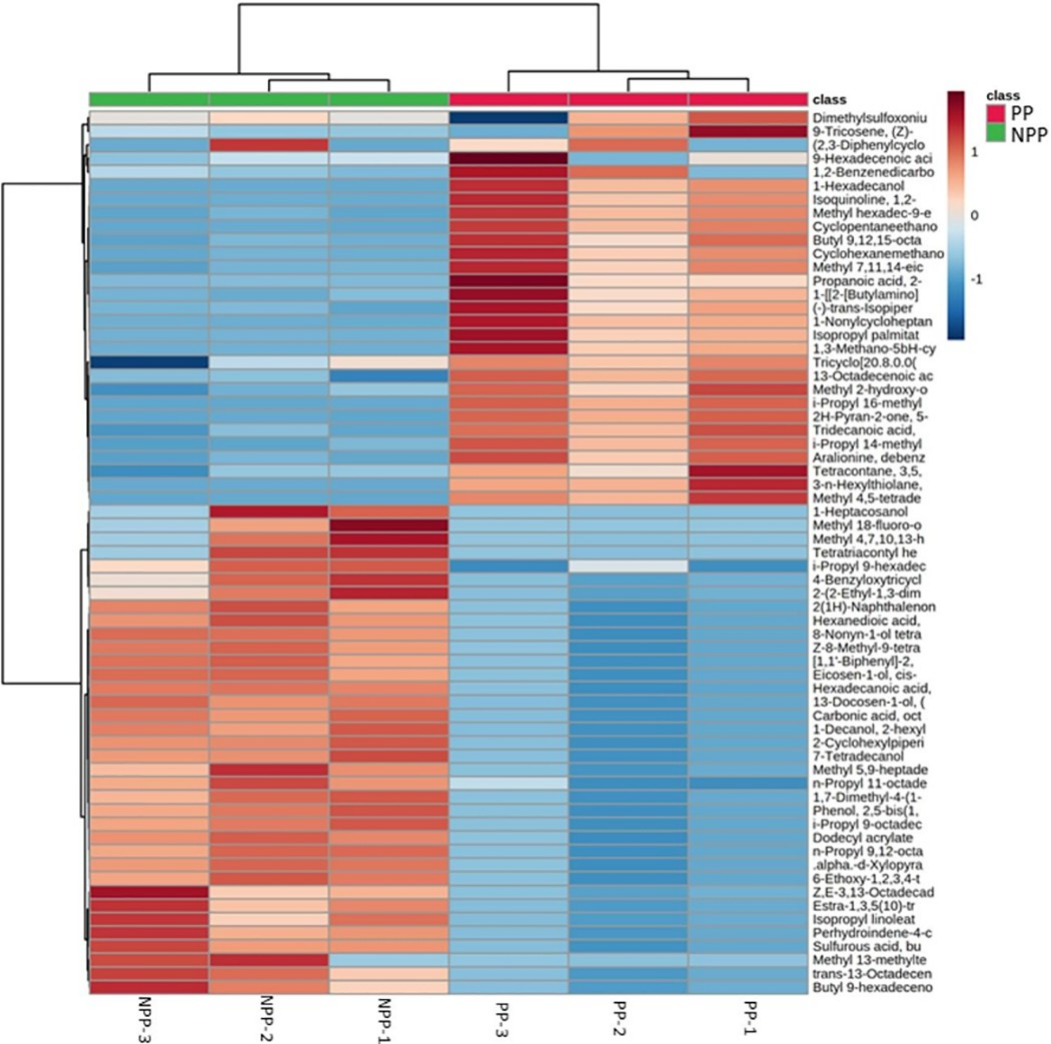
Hierarchical cluster analysis of medfly pupae metabolic profile under probiotic supplementation (X-axis). The Y-axis represents individual metabolites that were identified and showed changes in abundance with respect to preceding and following time points (p < 0.05). Normalized signal intensities are visualized as a color spectrum in the heat maps. Red and blue represent high and low expression, respectively, of metabolites.

**Fig 7:**
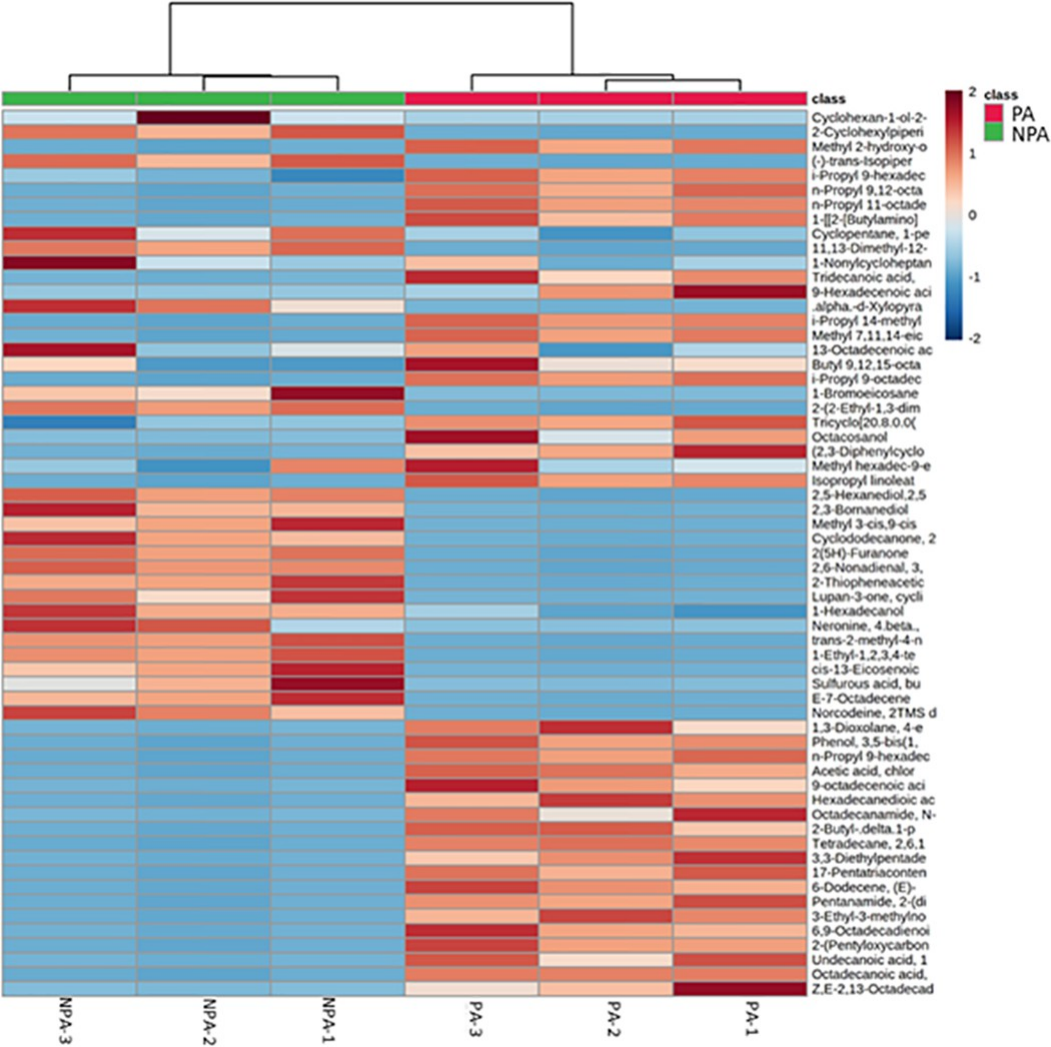
Hierarchical cluster analysis of medfly adults’ metabolic profile under probiotic supplementation (X-axis). The Y-axis represents individual metabolites that were identified and showed changes in abundance to preceding and following time points (p < 0.05). Normalized signal intensities are visualized as a color spectrum in the heat maps. Red and blue represent high and low expression, respectively, of metabolites.

Fluctuations in the different metabolites between the two groups were investigated, as visualized through the heatmaps showcased in Figs 5–7. Each heatmap comprises two blocks of metabolites, with the right block representing the metabolites of the medfly life stage supplemented with probiotics and the left block representing the metabolites of the non-probiotic medfly stage. These infographics clearly illustrate the pronounced disparities in metabolic profiles between the probiotic and non-probiotic stages of medfly, offering valuable insights into the potential impact of probiotics on the medfly’s metabolic pathways.

The majority of metabolites, including Aralionine, dibenzoyl;i-Propyl 14-methyl-pentadecanoate; Phenadoxone; Dimethylsulfoxoniumformylmethylide; Methyl hexadec-9-enoate (Methyl palmitoleate) and 1,2-Benzenedicarboxylic acid were increased in the probiotic larvae compared to non-probiotic larvae (Fig 5). In contrast, metabolites such as Z,E-3,13-Octadecadien-1-ol; 2-Cyclohexylpiperidine; 2(1H)-Naphthalenone,octahydro-4a-methyl-7-(1-methylethyl)-(4aalpha,7beta,8abeta) and (-)-Cis-Isopiperitenol were more abundant in non-probiotic larvae (Fig 5).

At the pupal stage, the probiotic supplementation led to a general reduction in the concentration of most metabolites compared to to non-probiotic pupae (Fig 6). Specifically, metabolites such as 2-Cyclohexylpiperidine;Z,E-3,13-Octadecadien-1-ol; Isopropyl 9Z-hexadecenoate (i-Propyl 9-hexadecenoate); Methyl(5Z,9Z)-heptadeca-5,9-dienoate; Perhydroindene-4-carboxylic acid, 6-acetoxy-2,3-epoxy-1,1-epoxymethyl-3a-hydroxy-5-isopropenyl-7a-methyl-7-oxo-, methyl ester; 4-Benzyloxytricyclo[4.3.1.0(3,8)]decan-10-ol; Methyl 4,7,10,13-hexadecatetraenoic acid; Methyl alpha-D-xylopyranoside (.alpha.-d-Xylopyranoside, methyl) and Isopropyl linoleate were decreased in probiotic pupae. Controversly, metabolites such as i-methyl-sulfoxonium-formyl-methylide; 2(1H)-Naphthalenone, octahydro-4a-methyl-7-(1-methylethyl)-.(4aalpha,7beta,8abeta)-; Methyl hexadec-9-enoate (Methyl palmitoleate); 14-methyl-pentadecanoate; Aralionine, dibenzoyl-; Methyl 2-hydroxy-octadeca-9,12,15-trienoate and (-)-trans-Isopiperitenol were increased in non-probiotic pupae.

Moreover, the heatmap presented in Fig 7 highlighted significant fluctuations in metabolite levels between non-probiotic and probiotic adult medflies, suggesting the influence of probiotic supplementation. Indeed, a lower concentration of some metabolites such as Z,E-3,13-Octadecadien-1-o; Isopropyl 9Z-hexadecenoate (i-Propyl 9-hexadecenoate); i-Propyl 14-methyl-pentadecanoate; and 1,3-Dioxolane, 4-ethyl-5-octyl-2,2-bis(trifluoromethyl)-, cis-. Conversely, a heightened level of several metabolites such as (-)-trans-Isopiperitenol;Methyl alpha-D-xylopyranoside (alpha.-d-Xylopyranoside, methyl); Norcodeine di-TMS derivative; 2,6-Nonadienal, 3,7-dimethyl; 2(5H)-Furanone; 2,3-Bornanediol and 2,5-dimethyl-2,5-Hexanediolin, was observed in non-probiotic adult medflies compared to adults after probiotic supplementation.

### Identification of potential biomarkers underlying the impact of probiotics in medfly metabolomic profile

Using VIP values greater than 1 and a p-value threshold of less than 0.05, we pinpointed a total of 7 upregulated and 5 downregulated metabolites for larvae supplemented with the probiotic strain. In adult medflies, 7 metabolites were upregulated while an equal number was downregulated. Conversely, for the case of probiotic pupae, all 12 metabolites displayed downregulation. Altogether, we identified 37 known metabolites, of which 14 demonstrated upregulation and 23 exhibited downregulation in the medfly life stages supplemented with probiotic strain (Table 1).

**Table 1:** Differentially expressed metabolites in the three medfly stages, larvae, pupae, and adults after probiotic supplementation.

Life stage	Compounds	VIP	FDR	FC	AUC	Sensitivity	Specificity
**PL** ***vs*** **NPL**	Dimethylsulfoxoniumformylmethylide	2.217	1.229E-4	4.0024	1.0	1	1
	2(1H)-Naphthalenone, octahydro-4a-methyl-7-(1-methylethyl)-. (4aalpha,7beta,8abeta)-	1.711	0.013	0.114	1.0	1	1
	Phenadoxone	5.571	1.7505E-4	4.3361	1.0	1	1
	2-Cyclohexylpiperidine	2.083	0.0087539	0.364	0.902	0.804	1
	Z,E-3,13-Octadecadien-1-ol	1.961	1.5893E-4	0.15482	0.877	0.854	0.9
	i-Propyl 9-hexadecenoate	1.549	1.4962E-4	3.7418	0.871	0.742	1
	Cyclopentane, 1-pentyl-2-prop	1.774	0.0045068	14.393	0.868	0.736	1
	Tridecanoic acid, 12-methyl-,	1.899	1.3273E-4	0.14295	0.851	0.902	0.8
	9-Hexadecenoic acid, methyl ester	3.874	0.0037116	0.12718	0.846	0.702	0.99
	i-Propyl 14-methyl-pentadecan	1.431	0.001157	2.2285	0.827	0.772	0.972
	Aralionine, debenzoyl-	1.098	0.0022497	4.4685	0.773	0.696	0.85
	1,2-Benzenedicarboxylic acid,	1.55	9.7568E-4	3.9776	0.745	0.592	0.898
**PP** ***vs*** **NPP**	2(1H)-Naphthalenone, octahydro-4a-methyl-7-(1-methylethyl)-. (4aalpha,7beta,8abeta)-	2.16	8.5534E-4	0.47	1.0	1	1
	1,2-Dipalmitoyl-rac-glycerol (Hexadecanoic acid, 1-(hydroxymethyl)-1,2-ethanediyl ester)	1.23	1.5893E-4	0.46	0.98	0.96	1
	2H-Pyran, tetrahydro-2-(8-nonynyloxy)or (8-Nonyn-1-ol tetrahydropyran-)	2.29	3.2624E-4	3.59	0.755	0.6	0.9
	Methyl 2-hydroxy-octadeca-9,12,15-trienoate	2.32	0.006272	3.91	0.74	0.7	0.8
	(-)-trans-Isopiperitenol	1.38	0.015348	12.45	0.73	0.7	0,8
	Methyl (5Z,9Z)-heptadeca-5,9-dienoate	1.45	0.0038862	0.43	0.72	0.6	0.8
	Perhydroindene-4-carboxylic acid, 6-acetoxy-2,3-epoxy-1,1-epoxymethyl-3a-hydroxy-5-isopropenyl-7a-methyl-7-oxo-, methyl ester	3.98	0.0023972	0.15	0.715	0.7	0.73
	4-Benzyloxytricyclo[4.3.1.0(3,8)]decan-10-ol	2.48	0.014445	5.88	0.715	0.55	0.85
	Methyl 4,7,10,13-hexadecatetraenoic acid	1.32	0.010319	4.46	0.71	0.6	0.8
	Z,E-3,13-Octadecadien-1-ol	2.09	0.015222	0.36	0.71	0.9	0.6
	i-Propyl 9-hexadecenoate	1,54	0.022493	20.14	0.7	0.6	0.9
	3-Deoxyestradiol or (Estra-1,3,5(10)-trien-17.beta)	1.42	0.0039493	0.37	0.7	0.55	0.85
**PA** ***vs*** **NPA.**	Norcodeine, 2TMS derivative	4.43	0.0020073	0.12168	1.0	1	1
	2(5H)-Furanone	1.45	7.7021E-5	0.16167	1.0	1	1
	2,5-Hexanediol,2,5-dimethyl-	2.11	1.0779E-4	6.2389	0.9844	0.938	1
	3Z,9Z,12Z-Octadecatrienoic acid	1.27	0.0051867	0.12524	0.89062	0.772	1
	1,3-Dioxolane, 4-ethyl-5-octyl-2,2-bis(trifluoromethyl)-, cis-	2.49	0.0094336	6.1964	0.8852	0.836	1
	(-)-trans-Isopiperitenol	3.84	9.0269E-4	0.14827	0.14827	0.725	0.921
	i-Propyl 9-hexadecenoate	6.43	0.001333	4.2752	0.82	0.709	0.916
	n-Propyl 9,12-octadecadienoate	3.06	2.5713E-4	4.279	0.79	0.684	0.906
	Methyl alpha-D-xylopyranoside	1.32	0.013728	0.09196	0.78158	0.654	0.906
	11,13-Dimethyl-12-tetradecen-1-ol acetate	3.11	1.127E-4	0.16358	0.77961	0.649	0.891
	Isopropyl linoleate	6.03	2.0208E-4	4.2342	0.76974	0.657	0876
	2,6-Nonadienal, 3,7-dimethyl-	2.17	8.7526E-5	7.1269	0.75395	0.607	0.896
	2,3-Bornanediol	1.68	0.0061987	0.12949	0.63281	0.632	0.895

To identify potential biomarkers associated with probiotic supplementation in medfly life stages, we selected relevant metabolites based on an area under the curve (AUC) greater than 0.7 and a false discovery rate (FDR) of less than 0.05. Following all extraction methods, we identified a total of 12 differential metabolites as potential biomarkers for the larval stage, 12 for the pupal stage, and 13 for the adult stage. The results were summarized in Table 1.

## Discussion

Our study aimed to assess the metabolic changes in medflies following probiotic supplementation. Using advanced PCA and OPLS-DA models, we analyzed the metabolic profiles of different medfly stages, including those treated with probiotics, non-treated stages, and the probiotic strain itself. The models demonstrated significant differences between treatments, with probiotic-treated stages and the probiotic strain standing out from non-probiotic stages. A total of 37 known metabolites were identified, of which 14 were upregulated and 23 downregulated. These metabolites were categorized based on their chemical structures, with lipids emerging as the predominant group. Notably, our heatmap analysis depicted clear and significant differences in the metabolic profiles between probiotic and non-probiotic stages, underscoring the profound impact of probiotic supplementation on medfly.

The study found that the probiotics (*Enterobacter* sp.) significantly influenced metabolites with antibacterial, antifungal and pheromone activity (S1 Table). Specifically, pheromones were down-regulated during the larval and pupal stages, but up-regulated in adult probiotic-treated individuals compared to their non-treated counterparts. These findings align with previous research indicating that the addition of probiotics to the diet improves the sexual competitiveness of males, enhances defenses against pathogens, and reduces copulation latency time within medfly [4,5,10,21,22].

Pheromones play a pivotal role in insect communication, influencing social behavior throughout all developmental stages including mate selection, locating oviposition sites, and foraging for food [23,24]. Pheromones are typically a mixture of various chemical compounds, including esters, acids, alkanes, and terpenes. While their composition is genetically determined, it is also subject to modulation by environmental factors such as temperature, humidity, population density, and dietary influences [25].

The composition of the pheromone mixtures has been the subject of extensive research, identifying key constituents such as geranyl acetate, (E,E)-α-farnesene, (E)-3-octanoic ethyl ester, and 2-ethylhexanoic acid [26,27]. Differences in male pheromone mixtures documented in different studies may stem in several factors, such as differences in volatile collection techniques, dietary influences, insect laboratory strains, and the sourcing of pheromones from wild populations. It has been shown that the pheromones released by laboratory adult males differ both qualitatively and quantitatively from those released by wild-type medflies [28]. (Z)-9-octadecenamide has been identified as a specific pheromone released by laboratory medflies [26]. Our study demonstrated that probiotic supplementation led to significant changes in the metabolites profile of male medfly. The findings imply that probiotics may have potential for the improvement of the SIT as a control method. There is currently limited research on the specific impact of probiotics on pheromone synthesis. Niyazi et al. [21] demonstrated that the probiotic supplementation did not affect pheromone-calling activity of male medflies. However, emerging evidence suggests that probiotics can modulate the gut microbiome [4,5] which may, in turn, affect various physiological processes including, pheromone production [29]. Recently, researchers have proposed that the microbiome-gut-brain axis may include a overlooked olfactory component, referred to as a microbiome-olfaction-behavior pathway [30]. Pobiotics may indirectly influence pheromone production by altering the gut microbiota and stabilizing microbial communities, potentially enhancing the competitiveness of sterile males. For instance, rectal bacteria, mostly *Bacillus* species, have been isolated from the rectum of male oriental fruit fly (*Bactrocera dorsalis* (Hendel)) where they produce sex pheromones such as 2,3,5,6-tetramethylpyrazine (TTMP) and 2,3,5,6-trimethylpyrazine (TMP). Indeed, the metabolic activity of the DEMs (as listed in S1 Table) revealed several metabolites such as 2H-Pyran, tetrahydro-2-(8-nonynyloxy)- [31,32], Z,E-3,13-Octadecadien-1-ol [33–35], Z,E-3,13-Octadecadien-1-ol [33,36], Isopropyl 9Z-hexadecenoate, and Linoleic acid propyl ester [37] have been previously identified as sex pheromones and aggregation pheromones. Although aggregation pheromones attract both sexes to a calling site in order to induce group formation, in the case of the medfly "leks" [3], they may be as important as sex pheromones, which act only on one sex but to the same end. Many Diptera’s sex pheromones are hydrocarbons derived from fatty acids [38]. Notably, our study demonstrated that after probiotic supplementation, fatty acyls or lipids were the most abundant metabolite group in larvae, pupae, and adults. These fatty acids serve as precursors for a range of compounds, such as more unsaturated fatty acids, hydrocarbons, and pheromones, are formed from saturated and unsaturated fatty acids.

## Conclusion

In conclusion, the introduction of probiotics into the larval diet of medflies induced alterations in the metabolic profiles of various life stages. Distinct metabolites were pinpointed as biomarkers for larval, pupal, and adult stages, showcasing diverse activities encompassing antibacterial, antifungal, and sexual and aggregation pheromones. The observed upregulation of metabolites identified as pheromones represents a crucial finding, particularly for sterile males, as it heightens their competitiveness with wild females post-release. Looking ahead, these insights could pave the way for targeted interventions in insect pest management, offering a promising avenue for refining and optimizing sterile insect techniques through the strategic use of probiotics to enhance the overall effectiveness of control programs. Moreover, the use of Enterobacteriaceae in medfly mass-rearing is still under investigation, with concerns regarding handler safety and environmental risk yet to be fully addressed. Two key processes require consideration for safety: the incorporation of probiotics into the larval-rearing medium and the administration of probiotics to sterile adult males before release. Strict biosecurity measures, such as daily surface and equipment decontamination using specific disinfectants, as well as the control of airflow within production modules, would be difficult to implement and would result in additional costs. However, there is a growing interest in using the inactivated form of the probiotic, which acts as a prebiotic. This alternative is easier to manage and yields comparable results.

## Supporting information

S1 Table Metabolic activity of the differentially expressed metabolites. (DOCX)

S1 File (XLSX)
