# Supplementary material for: Exploring the metabolic changes of Ceratitis capitata Vienna 8 strain across three developmental stages through probiotic larval diet supplementation
Source: PLoS One. 2024 Dec 2;19(12):e0313894. doi: 10.1371/journal.pone.0313894 (PMC11611148; doi:10.1371/journal.pone.0313894)
Supplement: S1 Table — (DOCX) [file pone.0313894.s001.docx]

| **Chemical classes** | **Compound (DEMS)** | **m/z** | **Chemical structure** | **Activity** | **Reference** |
| --- | --- | --- | --- | --- | --- |
| **Larvae** |  |  |  |  |  |
| [Thiocarbonyl compounds](http://classyfire.wishartlab.com/tax_nodes/C0001198) | Dimethylsulfoxonium formylmethylide | 63/78/45 | MF:[C_4_H_8_O_2_S](https://pubchem.ncbi.nlm.nih.gov/#query=C4H8O2S)  MW : 120.17 g/mol | Antioxidant, antifungal, antibacterial and anticancer | [39]  [40]  [41] |
| [Organooxygen compounds](http://classyfire.wishartlab.com/tax_nodes/C0000323) | 2(1H)-Naphthalenone, octahydro-4a-methyl-7-(1-methylethyl)-, (4a alpha,7beta,8abeta)- | 81/67/95 | MF:  [C_14_H_24_O](https://pubchem.ncbi.nlm.nih.gov/#query=C14H24O)  MW: 208.34 g/mol | NAD | <https://pubchem.ncbi.nlm.nih.gov/compound/41133> |
| Piperidines | 2-Cyclohexyl piperidine | 84/56/85 | [C_11_H_21_N](https://pubchem.ncbi.nlm.nih.gov/#query=C11H21N)  167.29 g/mol | [Antitubercular, anticonvulsants, inhibitor of σ2 Receptor](https://www.sciencedirect.com/science/article/pii/S0968089605012022) | [42]  [43]  [44] |
| Fatty acyls | Z,E-3,13-Octadecadien-1-ol | 55/41/81 | [C_18_H_34_O](https://pubchem.ncbi.nlm.nih.gov/#query=C18H34O)  266.5 g/mol | Female Sex Pheromones | [33]  [34]  [35] |
| Fatty acyls | Isopropyl 9Z-hexadecenoate | 55/69/83 | [C_19_H_36_O_2_](https://pubchem.ncbi.nlm.nih.gov/#query=C19H36O2)  296.5 g/mol | Aggregation pheromone | [37] |
| Saturated hydrocarbons | 1-Pentyl-2-propylcyclopentane | 69/55/83 | [C_13_H_26_](https://pubchem.ncbi.nlm.nih.gov/#query=C13H26)  182.35 g/mol | Hepatoprotective, anti-diarrhea, cardiotonic, antihyperglycemic | [45] |
| Fatty acyls | Methyl 12-methyltridecanoate | 74/87/43 | [C_15_H_30_O_2_](https://pubchem.ncbi.nlm.nih.gov/#query=C15H30O2)  242.4 g/mol | [Antibacterial, Antioxidant and anticancer](http://grasasyaceites.revistas.csic.es/index.php/grasasyaceites/article/view/1734) | [46] |
| Fatty acyls | Methyl palmitoleate | 55/69/74 | [C_17_H_32_O_2_](https://pubchem.ncbi.nlm.nih.gov/#query=C17H32O2)  268.4 g/mol | [Antibacterial](https://www.sciencedirect.com/science/article/pii/S0014579305010124) | [47] |
| Fatty acyls | i-Propyl 14-methyl-pentadecanoate | 57/256/102 | [C_19_H_38_O_2_](https://pubchem.ncbi.nlm.nih.gov/#query=C19H38O2)  298.5 g/mol | [Anti-bacterial, anti-fungal, anti-oxidant, and anti-inflammatory](http://ir-library.ku.ac.ke/bitstream/handle/123456789/17924/Antimicrobia%20activity%20and......pdf?sequence=1) | [48]  [49] |
| Benzen and substitutes derivatives | Phenadoxone | 114/115/56 | [C_23_H_29_NO_2_](https://pubchem.ncbi.nlm.nih.gov/#query=C23H29NO2)  351.5 g/mol | Analgesic, anti‐inflammatory, antioxidant, antiobesity, and antihyperlipidemic to antimicrobial, antineurodegenerative and anticancer. | [50] |
| Carboxylic acids and derivatives | Aralionine, debenzoyl- | 114/115/85 | [C_27_H_34_N_4_O_4_](https://pubchem.ncbi.nlm.nih.gov/#query=C27H34N4O4)  478.6 g/mol | NAD | <https://pubchem.ncbi.nlm.nih.gov/compound/5370466> |
| **Pupae** |  |  |  |  |  |
| [Organooxygen compounds](http://classyfire.wishartlab.com/tax_nodes/C0000323) | 2(1H)-Naphthalenone, octahydro-4a-methyl-7-(1-methylethyl)-. (4aalpha,7beta,8abeta)- | 81/67/95 | MF:  [C_14_H_24_O](https://pubchem.ncbi.nlm.nih.gov/#query=C14H24O)  MW: 208.34 g/mol | NAD | <https://pubchem.ncbi.nlm.nih.gov/compound/41133> |
| Glycerolipids | 1,2-Dipalmitoyl-rac-glycerol | 43/57/55 | [C_35_H_68_O_5_](https://pubchem.ncbi.nlm.nih.gov/#query=C35H68O5)  568.9 g/mol | Hydrolase activity | http://www.ymdb.ca/compounds/YMDB00834 |
| Oxanes | 2H-Pyran, tetrahydro-2-(8-nonynyloxy)- | 85/101/41 | [C_14_H_24_O_2_](https://pubchem.ncbi.nlm.nih.gov/#query=C14H24O2)  224.34 g/mol | Insect Sex attractant | [31]  [32] |
| Fatty Acyls | Methyl 2-hydroxy-octadeca-9,12,15-trienoate | 79/67/95 | [C_19_H_32_O_3_](https://pubchem.ncbi.nlm.nih.gov/#query=C19H32O3)  308.5 g/mol | Antioxidant activity | [51] |
| Prenol Lipids | (-)-trans-Isopiperitenol | 84/83/41 | [C_10_H_16_O](https://pubchem.ncbi.nlm.nih.gov/#query=C10H16O)  152.23 g/mol | Biosynthesis of monoterpene in leaves | https://www.uniprot.org/uniprot/Q5C9I9 |
| Fatty Acyls | Methyl (5Z,9Z)-heptadeca-5,9-dienoate | 81/109/69 | [C_18_H_32_O_2_](https://pubchem.ncbi.nlm.nih.gov/#query=C18H32O2)  280.4 g/mol | NAD | <https://pubchem.ncbi.nlm.nih.gov/compound/91697706> |
| Benzene and Substituted derivatives | 4-Benzyloxytricyclo[4.3.1.0(3,8)]decan-10-ol | 91/92/167 | [C_17_H_22_O_2_](https://pubchem.ncbi.nlm.nih.gov/#query=C17H22O2)  258.35 g/mol | NAD | <https://pubchem.ncbi.nlm.nih.gov/compound/561887> |
| Fatty Acyls | 4,7,10,13-hexadecatetraenoic acid | 5/41/131 | [C_16_H_26_O_2_](https://pubchem.ncbi.nlm.nih.gov/#query=C16H26O2)  250.38 g/mol | Anti-cancer | [52] |
| Faty Acyls | Z,E-3,13-Octadecadien-1-ol | 55/41/81 | [C_18_H_34_O](https://pubchem.ncbi.nlm.nih.gov/#query=C18H34O)  266.5 g/mol | Pheromones | [33]  [36] |
| Fatty Acyls | Isopropyl 9Z-hexadecenoate | 55/69/83 | [C_19_H_36_O_2_](https://pubchem.ncbi.nlm.nih.gov/#query=C19H36O2)  296.5 g/mol | Pheromones | [37] |
| Steroids and steroid derivatives | 3-Deoxyestradiol (Nom au tableau: Estra-1,3,5(10)-trien-17.beta) | 57/69/104 | [C_18_H_24_O](https://pubchem.ncbi.nlm.nih.gov/#query=C18H24O)  256.4 g/mol | Nephroprotective | [53] |
| **Adults** |  |  |  |  |  |
| Fatty Acyls | Methyl 2-hydroxy-octadeca-9,12,15-trienoate | 79/67/95 | [C_19_H_32_O_3_](https://pubchem.ncbi.nlm.nih.gov/#query=C19H32O3)  308.5 g/mol | Cannabinoid  antioxidant | <https://patentscope.wipo.int/search/en/result.jsf?inchikey=DYFXKQIZAHPOSD-YSTUJMKBSA-N>  <https://pubchem.ncbi.nlm.nih.gov/compound/91697556#section=Literature> |
| Prenol Lipids | [(-)-trans-Isopiperitenol](https://www.ncbi.nlm.nih.gov/pcsubstance/?term=%22(-)-trans-Isopiperitenol%22%5bCompleteSynonym%5d%20AND%20439410%5bStandardizedCID%5d) | 84/83/41 | [C_10_H_16_O](https://pubchem.ncbi.nlm.nih.gov/#query=C10H16O)  152.23 g/mol | Cannabinoid and flavone | <https://pubchem.ncbi.nlm.nih.gov/compound/439410#section=Patents> |
| Fatty Acyls | Isopropyl 9Z-hexadecenoate | 55/69/83 | [C_19_H_36_O_2_](https://pubchem.ncbi.nlm.nih.gov/#query=C19H36O2)  296.5 g/mol | Aggregation pheromone | [37] |
| Fatty Acyls | Linoleic acid propyl ester | 67/81/95 | [C_21_H_38_O_2_](https://pubchem.ncbi.nlm.nih.gov/#query=C21H38O2)  322.5 g/mol | Aggregation pheromone | [37] |
| Organooxygen compounds | Methyl alpha-D-xylopyranoside |  | [C_6_H_12_O_5_](https://pubchem.ncbi.nlm.nih.gov/#query=C6H12O5)  164.16 g/mol | [Stimulate Muscle](https://pubmed.ncbi.nlm.nih.gov/19049348) | [54] |
| Fatty Acyls | 11,13-Dimethyl-12-tetradecen-1-ol acetate | 69/43/95 | [C_18_H_34_O_2_](https://pubchem.ncbi.nlm.nih.gov/#query=C18H34O2)  282.5 g/mol | Antioxidant | [55] |
| Fatty Acyls | Isopropyl linoleate | 67/43/41 | [C_21_H_38_O_2_](https://pubchem.ncbi.nlm.nih.gov/#query=C21H38O2)  322.5 g/mol | Volatile compounds with high antioxidant activity | [56] |
| Organooxygen compounds | 2,5-Dimethyl-2,5-Hexanediol | 43/49/70 | [C_8_H_18_O_2_](https://pubchem.ncbi.nlm.nih.gov/#query=C8H18O2)  146.23 g/mol | Anti-cancer and toxic | [57] |
| Prenol Lipids | 2,3-Bornanediol | 95/43/41 | [C_10_H_18_O_2_](https://pubchem.ncbi.nlm.nih.gov/#query=C10H18O2)  170.25 g/mol | Sex attractant female pheromone (pesticide) | [58]  NCBI (2024)  [59] |
| Fatty Acyls | 3Z,9Z,12Z-Octadecatrienoic acid | 73/89/151 | [C_18_H_30_O_2_](https://pubchem.ncbi.nlm.nih.gov/#query=C18H30O2)  278.4 g/mol | Antioxidant | [60] |
| Dihydrofurans | 2(5H)-Furanone | 55/84/27 | [C_4_H_4_O_2_](https://pubchem.ncbi.nlm.nih.gov/#query=C4H4O2)  84.07 g/mol | Antimicrobial | [61] |
| Prenol Lipids | 2,6-Nonadienal, 3,7-dimethyl- | 55/41/83 | [C_11_H_18_O](https://pubchem.ncbi.nlm.nih.gov/#query=C11H18O)  166.26 g/mol | Antimicrobial | [62] |
| Morphinans | Norcodeine di-TMS derivative | 73/254/429 | [C_23_H_35_NO_3_Si_2_](https://pubchem.ncbi.nlm.nih.gov/#query=C23H35NO3Si2)  429.7 | NAD | https://pubchem.ncbi.nlm.nih.gov/compound/553624 |
| Organooxygen compounds | 1,3-Dioxolane, 4-ethyl-5-octyl-2,2-bis(trifluoromethyl)-, cis- | 69/97/83 | [C_15_H_24_F_6_O_2_](https://pubchem.ncbi.nlm.nih.gov/#query=C15H24F6O2)  350.34 | NAD | https://pubchem.ncbi.nlm.nih.gov/compound/91694991 |

NAD: Not determined activity

**References:**

39. Naine SJ, Devi CS, Mohanasrinivasan V, Vaishnavi B. Bioactive Potential of Marine Derived Strain *Streptomyces brasiliensis* VITJS9 Isolated from South East Coast of Tamil Nadu, India. Natl Acad Sci Lett. 2015; 38 (3) : 221-224.

40. Paudel MR, Chand MB, Pant B Pant, B. Assessment of Antioxidant and Cytotoxic Activities of Extracts of *Dendrobium crepidatum*. Biomolecules. 2019; 9 : 478.

41. Husein HA, Alhasan DAH, Albadry MAZ. In Vitro Antimicrobial Activity and GCMS Analysis of Crude Aqueous Methanolic Extract Produced from Leaves of Eucalyptus species. TQMJ. 2019: 17(1) : 54–69.

42. Ho B, Crider AM, Stables JP. Synthesis and structure–activity relationships of potential anticonvulsants based on 2-piperidinecarboxylic acid and related pharmacophores. Eur. J. Med. Chem. 2001; 36 : 265-286.

43. Kolavi G, Hegde V, Khazi Ia, Gadad P. Synthesis and evaluation of antitubercular activity of imidazo[2,1-b][1,3,4]thiadiazole derivatives. Bioorg Med Chem. 2006; 14 : 3069-80.

44. Berardi F, Abate C, Ferorelli S, Uricchio V, Colabufo NA, Niso M, Perrone R. Exploring the importance of piperazine N-atoms for sigma(2) receptor affinity and activity in a series of analogs of 1-cyclohexyl-4-[3-(5-methoxy-1,2,3,4-tetrahydronaphthalen-1-yl)propyl]piperazine (PB28). J Med Chem*.*2009; 52 : 7817–28.

45. Zailani MA, Badruddin Ahmad F. The Oils from Stems, Leaves and Roots of *Elephantopus scaber* Linn. BJRST [Internet]. 2016; 6(1) : 46-9.

46. National Center for Biotechnology Information (2023a). PubChem Patent Summary for CN-114796021-A. Retrieved November 9, 2023 from <https://patents.google.com/patent/CN114796021A/en>

47. Zheng CJ, Yoo JS, Lee TG, Cho HY, Kim YH, Kim WG. Fatty acid synthesis is a target for antibacterial activity of unsaturated fatty acids. FEBS Lett. 2005; 26; 579(23) : 5157-62.

48. Junairiah J, Nurhariyati T, Ni’matuzahroh, Sulistyorini L. Isolation of bioactive compounds from *Dicranaceae Mosses*. J. K. R. 2016; 1(2) : 11-121.

49. Otieno A J. 2016. Antimicrobial activity and phytochemical profiles of *warburgia ugandensis* Sprague (Canellaceae) extracts from different populations across the Kenyan Rift Valley. Thesis 214p.

50. National Center for Biotechnology Information (2023b). PubChem Patent Summary for WO-2022241186-A1, Compositions for and methods of treating and/or preventing pain. Retrieved November 9, 2023 from <https://patents.google.com/patent/WO2022241186A1/fr?oq=WO-2022241186-A1>

51. Chan HWS, Levett G. Autoxidation of methyl linoleate. Separation and analysis of isomeric mixtures of methyl linoleate hydroperoxides and methyl hydroxylinoleates. Lipids, 1977. 12 : 99-104.

52. National Center for Biotechnology Information (2023c). PubChem Patent Summary for CN-114788872-A. Retrieved November 9, 2023 from  <https://patents.google.com/patent/CN114788872A/en?oq=CN-114788872-A>

53. Sohail N, Hira K, Kori JA, Farhat H, Urooj F, Khan W, Sultana V, Ali MS, Ehteshamul-Haque S*.* Nephroprotective effect of ethanol extract and fractions of a sea lettuce, *Ulva fasciata* against cisplatin-induced kidney injury in rats. Environ Sci Pollut Res*.* 2021; 28 : 9448–9461.

54. Gruzman A, Shamni O, Ben Yakir M, Sandovski D, Elgart A, Alpert E, Cohen G, Hoffman A, Katzhendler Y, Cerasi E, Sasson S. Novel D-xylose derivatives stimulate muscle glucose uptake by activating AMP-activated protein kinase alpha. J Med Chem. 2008; 51(24) : 8096-108.

55. Ningsih R, Rafi M, Tjahjoleksono A, Bintang M, Megia R. Ripe pulp metabolite profiling of ten Indonesian dessert banana cultivars using UHPLC-Q-Orbitrap HRMS. Eur Food Res Technol. 2021; 247 : 2821–2830.

56. Kim BR, Kim HM, Jin CH, Kang SY, Kim JB, Jeon YG, Park KY, Lee IS, Han AR. Composition and Antioxidant Activities of Volatile Organic Compounds in Radiation-Bred Coreopsis Cultivars. Plants (Basel). 2020; 9(6) : 717.

57. Hunt WA, Majchrowicz E. Suppression of the ethanol withdrawal syndrome by aliphatic diols. ASPET. 1980; 213 : 9-12

58. Lower SE, Pask GM, Arriola K, Halloran S, Holmes H, Halley D, Zhzng Y, Collins D, Millar J*.* Identification of a Female-Produced Sex Attractant Pheromone of the Winter Firefly, *Photinus corruscus* Linnaeus (Coleoptera: Lampyridae). J Chem Ecol. 2023; 49 : 164–178.

59. National Center for Biotechnology Information (2024). PubChem Patent Summary for WO-2019180420-A1, Pesticide. Retrieved January 3, 2024 from <https://pubchem.ncbi.nlm.nih.gov/patent/WO-2019180420-A1>

60. Tsevegsuren N, Fujimoto K, Christie WW, Endo Y. Occurrence of a novel cis,cis,cis-octadeca-3,9,12-trienoic (Z,Z,Z-octadeca-3,9,12-trienoic) acid in *Chrysanthemum* (tanacetum) *zawadskii* herb. (Compositae) seed oil. Lipids. 2003; 38(5) : 573-8.

61. Khabibrakhmanova AM, Faizova RG, Lodochnikova OA, Zamalieva RR, Latypova LZ, Trizna EY, Porfiryev AG, Tanaka K, Sachenkov OA, Kayumov AR, Kurbangalieva AR. The Novel Chiral 2(5H)-Furanone Sulfones Possessing Terpene Moiety: Synthesis and Biological Activity. Molecules. 2023; 28(6) : 2543.

62. Li M, Han G, Chen H,Yu J, Zhang Y. Chemical compounds and antimicrobial activity of volatile oils from bast and fibers of *Apocynum venetum* . Fibers Polym*.* 2012; 13 : 322–328.
